# Addressing inactivity after stroke: The Collaborative Rehabilitation in Acute Stroke (CREATE) study

**DOI:** 10.1177/1747493020969367

**Published:** 2020-11-02

**Authors:** Fiona Jones, Karolina Gombert-, Stephanie Honey, Geoffrey Cloud, Ruth Harris, Alastair Macdonald, Christopher McKevitt, Glenn Robert, David Clarke

**Affiliations:** 1 Faculty of Health, Social Care and Education, Kingston University & St George’s, University of London, London, UK; 2 Leeds Institute of Health Sciences, University of Leeds, Leeds, UK; 3 Alfred Health, Melbourne, Australia; 4 Department of Clinical Neurosciences, Central Clinical School, Monash University, Melbourne, Australia; 5 Faculty of Nursing, Midwifery and Palliative Care, King’s College London, London, UK; 6 School of Design, Glasgow School of Art, Glasgow, UK; 7 Faculty of Life Sciences and Medicine, School of Population Health & Environmental Sciences, King’s College London, London, UK

**Keywords:** Stroke, inactivity, co-design

## Abstract

**Background:**

Stroke patients are often inactive outside of structured therapy sessions – an enduring international challenge despite large scale organizational changes, national guidelines and performance targets. We examined whether experienced-based co-design (EBCD) – an improvement methodology – could address inactivity in stroke units.

**Aims:**

To evaluate the feasibility and impact of patients, carers, and staff co-designing and implementing improvements to increase supervised and independent therapeutic patient activity in stroke units and to compare use of full and accelerated EBCD cycles.

**Methods:**

Mixed-methods case comparison in four stroke units in England.

**Results:**

Interviews were held with 156 patients, staff, and carers in total; ethnographic observations for 364 hours, behavioral mapping of 68 patients, and self-report surveys from 179 patients, pre- and post-implementation of EBCD improvement cycles.

Three priority areas emerged: (1) ‘Space’ (environment); (2) ‘Activity opportunities’ and (3) ‘Communication’. More than 40 improvements were co-designed and implemented to address these priorities across participating units. Post-implementation interview and ethnographic observational data confirmed use of new social spaces and increased activity opportunities. However, staff interactions remained largely task-driven with limited focus on enabling patient activity. Behavioral mapping indicated some increases in social, cognitive, and physical activity post-implementation, but was variable across sites. Survey responses rates were low at 12–38% and inconclusive.

**Conclusion:**

It was feasible to implement EBCD in stroke units. This resulted in multiple improvements in stroke unit environments and increased activity opportunities but minimal change in recorded activity levels. There was no discernible difference in experience or outcome between full and accelerated EBCD; this methodology could be used across hospital stroke units to assist staff and other stakeholders to co-design and implement improvement plans.

## Introduction

Evidence that increasing the frequency and intensity of stroke rehabilitation can improve outcomes has driven numerous international guidelines and other major developments in hospital-based stroke care to achieve larger doses of therapy provided over seven days.^1,2^ However, outside of the scheduled therapy, inactivity is common and observational studies show stroke patients can be inactive and alone for more than 60% of waking hours, an issue largely unchanged for decades.^3,4^

There is now more understanding that rehabilitation intensity and outcomes cannot be improved by national targets alone – the stroke unit environment and how time is spent outside of scheduled face-to-face therapy are of critical consideration. Attempts to address inactivity have had mixed results. Dose-driven interventions including circuit class therapy and seven-day therapy have increased therapy provision but not patient activity outside of sessions.^5^ Some progress has been made by applying environmental enrichment evidence from animal models.^6^ Studies conducted in Australia have utilized controlled pre- and post-designs and evaluated the impact of more stimulating environments on inpatient activity.^3,7^ Behavior mapping showed an increase in activity levels across all domains and some changes were sustained at six months post intervention. However, the environmental enrichment was driven by the perspectives of researchers and professionals without patient and carer involvement and no specific quality improvement (QI) methodology. Improvement research is now recognized to be critical to ‘cumulate, synthesize and scale learning’ to expedite the translation of evidence into practice.^8^ We believed that a robust QI methodology could address the intractable issue of patient inactivity.

Across healthcare internationally, there is increasing evidence of improvement methodologies which involve patients and staff working collaboratively to help co-design solutions and deliver healthcare services.^9^ Experience-based co-design (EBCD) is an approach which enables staff and patients to co-design services in partnership. Experiences are gathered from patients and staff through in-depth interviewing, observations and group discussions, to identify key ‘touch points’ or emotionally positive or negative issues. An edited ‘trigger’ film is created from patient interviews to convey experiences of the service. Staff and patients are then brought together to explore the findings and to work in small groups to identify, co-design and implement activities that will improve the service or the care pathway.^10,11^ EBCD now has widespread use and led to improvements across multiple healthcare settings, including acute hospitals – but can lack detailed evaluation of feasibility and impact.^12^ To date EBCD has not been used as an improvement method in stroke units to address inactivity.

## Aims

The Collaborative Rehabilitation in Acute Stroke study (CREATE) aimed to (1) evaluate the feasibility of patients, carers and staff collaborating to develop and implement changes to increase supervised and independent therapeutic patient activity in acute stroke units; and (2) understand if improvements developed by two initial stroke units could be transferred to two further units and implemented within a shortened time frame using an accelerated form of EBCD (AEBCD).

**Figure 1.**
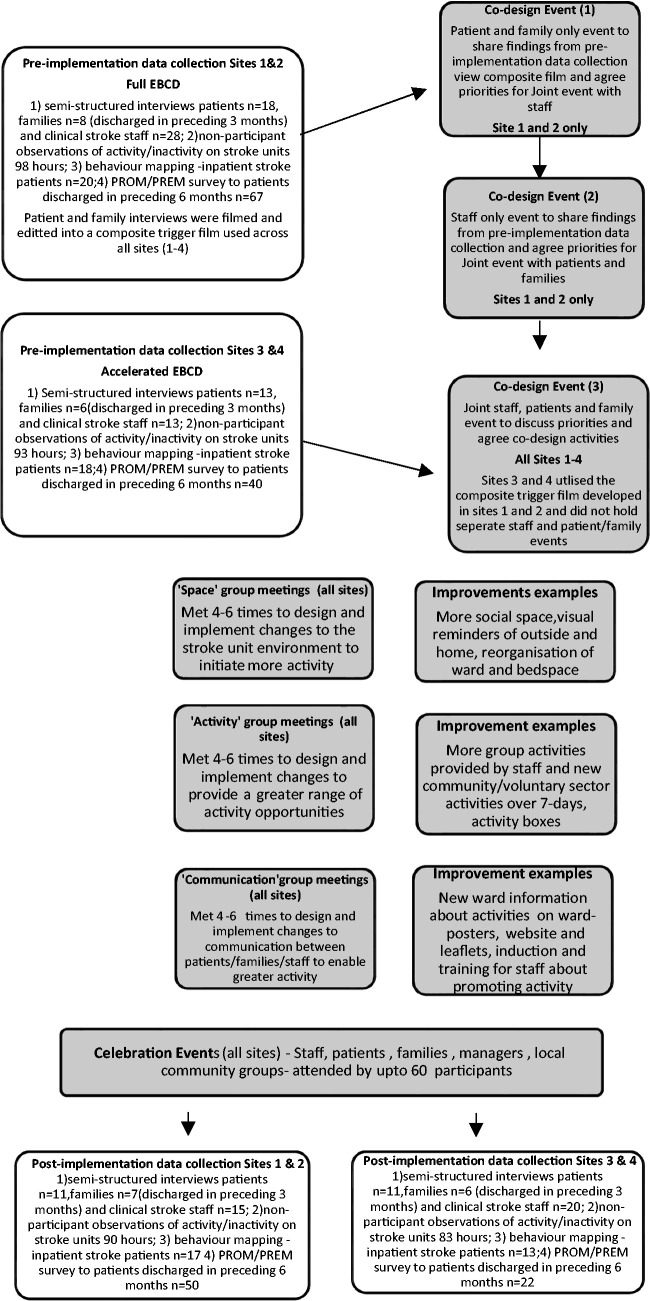
Showing accelerated and full experienced-based co-design with pre- and post-implementation data collection.

Figure 1 provides an overview of the stages of EBCD and AEBCD, data collected, and cohorts included pre- and post-implementation of improvements. Full EBCD and AEBCD took nine and six months to complete, respectively.

## Methods

### Design

A mixed-methods case study approach used to evaluate the feasibility and impact of patients, carers and staff co-designing and implement improvements to increase supervised and independent therapeutic patient activity in stroke units, and to compare use of full and accelerated EBCD cycles. A case study generates an in-depth, multi-faceted understanding of a complex issue in its real-life context. Comparisons pre- and post-implementation or between sites can be made but any inferences about causality are limited.^13^

We carried out pre- and post-implementation interviews (staff, patients, and families), qualitative ethnographic observations, and behavioral mapping, and utilized patient-reported outcome and experience measures to explore each site as a separate ‘case’. Data were collected prior to and following each EBCD/AEBCD phase activity. An embedded process evaluation drawing on Normalization Process Theory (NPT) considered barriers and facilitators to the use of EBCD/AEBCD to develop and implement the improvements in the study settings, and is reported separately.^14^

### Improvement mechanism

EBCD or AEBCD was introduced into all four stroke units. Sites 1 and 2 (S1 and S2) employed all six stages of EBCD to co-design improvements to address stroke patients’ physical, social, and cognitive activity. In sites 3 and 4 (S3 and S4), they commenced with a joint staff, patient and family member event to immediately agree improvement priorities and initiate co-design work prompted by trigger films previously developed in S1 and S2. In all four sites, smaller co-design groups (*n = *6–10) were attended by equal numbers of patients, families and stroke staff, jointly developing and implementing actions to address priority areas. Work to implement the agreed actions was shared based on existing skills, interest/opportunity and time available.

### Setting

Four stroke units based in hospitals in London and Yorkshire. National (England) acute stroke organizational data, reported bi-annually, showed that all four units performed within the mid-range across key quality indicators and were subject to common staffing pressures and increasing caseload complexity.

## Data Sources

1. **Semi-structured interviews with patients and carers** were conducted who were recruited through former inpatients, within three months of discharge (community-dwelling stroke survivors). Filmed narrative interviews were completed with consent from former inpatients and their carers from S1 and S2 to elicit perceptions and recall of opportunities for and experiences of activity in the stroke units. Films were edited to produce composite trigger films of 18–20 minutes duration.
2. **Semi-structured interviews were conducted with clinical stroke staff** who were recruited purposively to include a range of seniority and experience including professional and support staff. Interviews were used to explore their perceptions of the stroke unit environment and opportunities for and experiences of patient activity.
3. **Patient reported outcome (PROM) and patient reported experience measures (PREM)** were sent to 60 consecutive patients cared for in each unit in the six months pre- and post-implementation of the (A)EBCD cycle. The PROM incorporates validated measures including the Oxford Handicap Scale, the Subjective Index of Physical and Social Outcome (SIPSO), and the EQ5D.^15–17^ The PREM was the ‘Neurological Experience questionnaire’ published by Kneebone et al. (2012).^18^
4. **Ethnographic non-participant observations** used an observational framework developed in a previous evaluation process of stroke caregiver training^19^ and was used to record observations of stroke unit contexts, organizational processes, staff and patient interactions and instances of planned and unplanned activity, including noting when timetabled therapy was occurring on a one-to-one or group basis. Observations took place pre- and post-implementation of (A) EBCD across 10 days at different times including evenings and weekends.
5. **Behavioral mapping** (BM). Observers followed guidance for mapping according to Janssen et al.^20^ and tested reliability by comparing the results of two observers. Anomalies such as not directly observing patients in therapy or when outside the ward were discussed and the protocol adapted to ensure consistency. Participants were recruited if they were an inpatient on the stroke unit with a confirmed diagnosis of stroke and were able to provide informed consent the day before data collection. Mapping recorded social, cognitive, or physical activity levels in each site prior to separate or joint events and after celebration events. A comparison of activity levels in individual patients was not possible because their inpatient period did not span pre- and post-implementation data collection, and thus analysis served only as a broad indicator of activity level pre- and post-implementation of improvements. Patients were observed at 10-min intervals between 08:00 and 17:00 hours; or between 13:00 and 20:00 hours on three separate days, generating 60 observations for each patient per day. During each 10-min interval, the data for each patient was based on an observation of no longer than 5 seconds.

## Data analysis

Findings from the five data sources listed above informed the priority setting discussions by co-design groups in all four sites.

### Interview data

All data, including filmed patient interviews, were transcribed verbatim. NVIVO 11 software was used to manage and organize, label, and build coding categories. Analytical themes were generated through several iterative stages and involved reading and re-reading codes generating illustrative quotes and building categories, reviewing and refining final themes.^21^

### Observational data

Field notes were recorded during each observation, written in full immediately afterwards and summary memos produced. Data were subject to the same thematic analysis used for interview data.

### Behavioral mapping

All data were entered into an SPSS file and the frequency of occurrence of activity for each participant across each data collection period was summarized and used to generate descriptive statistics to quantify the proportion of physical, social and cognitive activity occurring for each patient during the period of observation. The objective was to record independent or supervised activity outside of therapy, so we took the decision to record scheduled therapy sessions as “unobserved”. “Unobserved” was also recorded when, for example, a patient was behind the bedside curtain or absent from the ward. “No activity” was only recorded when the patient was directly observed, their location was clear, and they were not engaged in activity.

**PREM and PROM data** were entered into SPSS and reported as descriptive statistics (or frequency counts) for each item for each site. These data quantified patients’ perceived functioning post stroke (PROM) and their reported experiences of the stroke unit (PREM).

## Results

EBCD was completed in S1 and S2, improvement priorities agreed, and changes implemented within nine months. AEBCD in S3 and S4 was completed and changes implemented within six months. Interview and observational data were also utilized for an embedded process evaluation which considered barriers and facilitators to implementation of the improvements in the study settings. Data sources and sample size are shown in Table 1, and demographic details of patient, staff and carer participants are shown in Tables 2 to 4. We did not collect individual data on stroke severity or mobility, but participants recruited for behavior mapping were beyond 72 h post stroke, not eligible to be discharged from their routinely admitting hyper acute stroke unit, consequently they included more non-ambulatory patients, than those recruited for interviews and co-design groups who were already discharged from the stroke unit and living in a community setting.

**Table 1.** Data collection pre and post implementation sites^1–4^

Data collection method	Site 1 pre	Site 1 post	Site 1 total	Site 2 pre	Site 2 post	Site 2 total	Site 3 pre	Site 3 post	Site 3 total	Site 4 pre	Site 4 post	Site 4 total	Median	Mean	All sites Total
Staff interviews (*n*)	13	8	21	15	7	22	6	8	14	7	12	19	n/a	n/a	76
Patient interviews (*n*)	9	5	14	9	6	15	9	6	15	4	5	9	n/a	n/a	53
Carer interviews (*n*)	4	5	9	4	2	6	3	3	6	3	3	6	n/a	n/a	27
PROM/PREMs	22	24	46	45	26	71	28	11	39	12	11	23	25	30	179
Behavior Mapping – participants (*n*)	9	7	16	11	10	21	12	7	19	6	6	12	10.5	11	68
Behaviour mapping – number of observations	702	949	1651	769	528	1297	945	782	1727	655	701	1356	863.5	1005.1	6031
Non-participant ethnographic observation (hours)	50	46	96	48	44	92	49	37	86	44	46	90	48.5	61	364

Note: Total number of Behaviour mapping observations generated every 10 min; Non-participant ethnographic observations generating qualitative data. All interviews were semi-structured; patient and carer interviews were filmed in sites 1 and 2 pre-implementation and edited into a composite ‘trigger film’; PROM: Patient Reported Outcome Measure; PREM: Patient Reported Experience Measure.

**Table 2.** Demographic details of patient participants

Variables/Sites	Site 1 BM (pre)	Site 1 Int (pre)	Site 1 BM (pr)	Site 1 Int (pr)	Site 2 BM (pre)	Site 2 Int (pre)	Site 2 BM (post)	Site 2 Int (post)	Site 3 BM (pre)	Site 3 Int (pre)	Site 3 BM (pr)	Site 3 Int (post)	Site 4 BM (pre)	Site 4 Int (pre)	Site 4 BM (post)	Site 4 Int (post)
Mean age (SD)	69 (12)	65 (12)	70 (18)	65 (13)	76 (14)	68 (18)	71 (17)	73 (12)	63 (15)	61 (16)	67 (16)	67 (18)	75 (12)	72 (7)	59 (9)	68 (10)
Females (%)	33	44	42	40	64	44	30	0	50	44	42	33	33	25	33	66
Mean time post stroke in days (SD)	24 (19)	72 (41)	49 (38)	65 (13)	26 (16)	68 (56)	30 (21)	52 (18)	27 (28)	58 (25)	25 (16)	40 (28)	44 (19)	85 (44)	35 (28)	64 (46)
Mean length of stay in days (SD)	35 (22)	50 (30)	55(22)	82 (52)	38 (20)	41 (32)	36 (28)	33 (18)	69 (50)	68(46)	42(26)	46 (13)	36 (24)	48 (18)	41 (22)	39 (26)
Aphasia (%)	33	22	29	40	9	22	40	17	25	11	29	33	67	75	75	50
Ethnicity	WB-7 Asian-1 BC-1	WB-5 Asian-1 BC-1	WB-3 Asian 2	WB-3 Asian-1 BC-1	WB = 11	WB = 9	WB = 10	WB = 6	WB-4 Asian-4 BC-4	WB-3 Asian-2 BC-3 BA-1	WB-2 Asian-2 BC-3	WB-2 Asian-1 BC-3	WB = 6	WB = 4	WB = 5	WB = 6

Note: We estimated all stroke participants to be moderate to severe strokes (in terms of approximate NIHSS classification), as stroke survivors with a mild stroke were discharged from the Hyper Acute Stroke Units either directly home or to an Early Supported Discharge Service; Sites did not consistently record Barthel or FIM and these were not recorded by the research team. All site participants had mobility limitations and the range was from walking with the standby supervision of one, use of a walking frame, walking with the assistance of one/two or wheelchair dependent; Ethnicity recorded using UK Ethnic grouping; WB: White British; W (other), W Irish, BC: Black Caribbean; BA: Black African; Asian, Mixed/Multi-ethnic groups; Other: ethnic Group.

**Table 3.** Staff participants

Variables/Sites	Site 1 (pre)	Site 1 (post)	Site 2 (pre)	Site 2 (post)	Site 3 (pre)	Site3 (post)	Site 4 (pre)	Site 4 (post)
Age								
18–24	0	0	1	0	0	0	0	0
25–34	4	3	9	5	1	2	2	4
35–44	5	4	4	1	3	3	2	2
45–54	4	1	1	1	2	3	2	2
55–64	0	0	0	0	0	–	1	1
Sex *N*, Females (%)	82	75	100	100	100	100	86	89
Profession:	AHP-5 RN-2 Psych-1 SW-3 Dr-1	AHP-4 RN-1 SW-1 Dr-1 GM-1	AHP-7 RN-3 SW-3 Dr-1 Vol-1	AHP-2 RN-2 SW-3	AHP-3 RN-1 Psych-1 Dr-1	AHP-3 RN-1 Psych-1 SW-2 Dr-1	AHP-3 RN-2 SW-1 Dr-1	AHP-3 RN-4 SW-2
Grade: % above Band 4	87	87	80	57	100	100	86	78
Ethnicity	WB-6 WI-1 W(other)-2 BC-1 BA-1 Asian-1	WB-4 WI-1 W(other)-2 Asian-1	WB-15	WB-7	WB-4 W(other)-1 Asian-1	WB-4 W(other)-1 Asian-3	WB-7	WB-9

Note: F female’ WB White British; W (other): any other White background; BC: Black Caribbean; BA: Black African; AHP: Allied Health Professional; Dr: Doctor; RN: Registered Nurse; Psych: Psychologist; SW: support worker; GM: General Manage; Vol: volunteer. Grade: Agenda for Change grading system for NHS staff (UK) which excludes Doctors, band 4 and below for technical and support staff, band 5 and above professional staff.

**Table 4.** Carer participants (semi-structured interviews)

Variables/Sites	Site 1 (pre)	Site1 (post)	Site 2(pre)	Site2 (post)	Site 3 (pre)	Site3 (post)	Site 4 (pre)	Site 4 (post)
Age								
18–24	1	1	0	0	0	1	0	0
25–34	0	0	0	0	0	0	0	0
35–44	0	0	0	0	0	0	0	0
45–54	1	1	0	0	0	0	0	0
55–64	2	1	1	0	1	0	1	2
65–74	0	1	2	0	1	2	1	2
75–84	0	1	1	2	1	0	0	0
85–94	0	0	0	0	0	0	0	1
Females (%)	50	40	75	100	100	33	50	80
Relationship to patient	2 × daughters, 1 grandson, 1 × partner	1 × son, 1 × daughter, 3 × partners	4 × partners	2 × partners	3 × partners	2 × partners 1 × sibling	2 × partners	5 × partners
Ethnicity	Asian-1 WB-3	Asian-1 WB-4	WB-4	WB-2	Asian-1 WB-2	Asian-1 WB-1 BC-1	WB-2	WB-5

Ethnicity; WB: White British; BC: Black Caribbean; BA: Black Africa; Asian; W (other): any other White background, multi-ethnic.

Pre-implementation observational data across all sites showed similar findings to previous research that patients spend much of their time inactive and disengaged. Analysis from observations (191 h) and interviews (*n* = 86) across all sites revealed a similar range of restrictions posed by the stroke unit environment, a culture of communication that was task-driven and limited opportunity for activity outside of scheduled therapy; these issues formed the priority areas for all co-design groups. Behavioral mapping data across sites revealed that between 0700 and 2000 hours, patients were inactive physically between 71% and 50%; cognitively inactive between 68% and 46% and socially inactive 58–31% of the day. Pre-implementation data and trigger films were shared with staff/former patients and carers at joint events which together acted as a mechanism of change for the formation of priorities and co-design groups. The outcomes from the EBCD/AEBCD cycles were multiple improvements in all stroke unit spaces/environments which led to provision of increased activity opportunities. An example of how pre-implementation data informed priority setting for co-design groups is given in Table 5, group characteristics in Table 6, and the number of co-design groups and an example of improvements implemented are shown in Table 7.

**Table 5.** Excerpts from analysis of field notes and interviews and how priorities were shaped

Priority	Example of limitations to activity
**Space** – “Restrictions to activity posed by the stroke unit environment”	“*When she (the carer’s wife) first went in…a really elderly lady was in the next bed and she couldn’t communicate at all. If they could have rotated the beds round so that the ones who could talk to each other [were next to each other], rather than have to talk over them to another patient, that would have been better for them”.* (Interview carer, site 2, pre)
**Activity** – “Limited opportunities for patients to be active outside of therapy”	*“I think [being a patient in here] it must be incredibly and utterly boring, I think,[…] there is the odd occasional therapy session from speech, OT and physio, I mean that would amount to maybe, what, two and a half hours, if that, maybe three.”* (Interview staff, site 2, pre)
**Communication** – “Driven by structures and routines not enabling to activity”	*“Healthcare assistant walks in and pushes her trolley next to him. She records some routine observations of pulse and blood pressure. They don’t talk. He closes his eyes while she performs the procedure.” (site 1)* (Observations, site 1, pre)

Note: Space, activity, and communication were priority areas agreed by patients, carers and staff at joint events. Quotes illustrate activity limitations from interview and ethnographic observational data which facilitated priority setting.

**Table 6.** Co-design group characteristics

Variables/Sites	Site 1 (*n* = 11)	Site2 (*n* = 8)	Site 3 (*n* = 18)	Site 4 (*n* = 7)
Age				
18–24	2	0	2	0
25–34	0	0	1	0
35–44	3	0	3	1
45–54	1	0	3	0
55–64	2	1	3	2
65–74	2	7	4	3
75–84	1	0	2	1
Females (%)	50	50	44	50
Ethnicity	WB-8 Asian-3 BC-1	WB-8	WB-8 Asian-3 BA-1 BC-6	WB-7
Non-ambulant stroke survivors (%)	36	50	27	33
Stroke survivors with aphasia (%)	27	25	22	66

Note: All stroke survivors participating in co-design groups were community dwelling; had been inpatients in the corresponding site within the last six months; Ethnicity; WB: White British; BC: Black Caribbean; BA: Black Africa; Asian, W (other): any other White background and Multi-ethnic.

**Table 7.** Co-design groups in each site

	Co design participants			Number of co-designs (group meetings)	Improvement examples (more examples are shown in supplementary files)
	PATIENTS (*n*)	CARERS (*n*)	STAFF (*n*)		
Site 1	5	6	13	10	New social space, creative workshops at weekends, therapy dog, access to tea/coffee making; bedside photo-hangers; activity boxes; volunteers, community groups
Site 2	4	4	12	9	Dayroom redesigned enabling use by patients and families; volunteer/staff led breakfast and lunch groups, film nights. Individualized social information boards for all patients. Choirs, reading support. Improved Wi-Fi access.
Site 3	11	7	14	15	New kitchen/social space for patients/families; weekend exercise and singing groups, information about unit ethos and personalising bed space; access to garden; new induction for staff to promote activity
Site 4	3	4	15	9	Room used for wheelchair storage repurposed and used for breakfast and social eating groups, quiet space for patients and families. Seating area to tend house plants, play board games, read magazines. Wi-Fi access promoted.
TOTAL	23	21	54	43	

Note: Improvement examples are those generated by co-design groups in priority areas to address Space, Activity and Communication; Co-design groups were jointly facilitated by staff, patients and researchers.

## Qualitative findings (interviews and observations)

Analysis of observational and interview data post-implementation of improvements confirmed discernible changes in the nature and use of communal ward spaces, more group activity, e.g. breakfast and art groups and increased activity opportunities at individual level. Patients and families had access to new social spaces to meet and interact, and organized groups and community volunteers. Stroke units communicated more consistently about activity opportunities through leaflets and posters, highlighting activities available outside of therapy and how patients and families could get involved. However, we found minimal change in the nature of face-to-face interactions between staff and patients outside of therapy, which remained task-focused with minimal interaction beyond that required for routine care tasks.

Overall staff, patients, and carers perceived that using the EBCD/AEBCD approach was associated with the improvements we report. Sites 1 and 2 together implemented more than 40 improvements across the three priority areas over nine months. Filmed patient narratives from S1 and S2 were perceived as powerful triggers for action and were utilized in S3 and S4 where a similar number and range of improvements were implemented over six months. Changes included those listed in Table 7 and mostly comprised environmental and organizational changes enabling greater social interaction between staff, patients and families. A number of additional improvements continued or were just getting underway as we ceased research involvement in sites including art courses and weekend activities groups provided by community groups. An example of perceived impact of changes is shown below in Table 8.

**Table 8.** Impact of co-designed changes

Priority	Improvement	Impact
**Space** – “Restrictions to activity posed by the stroke unit environment”	Space previously used to store wheelchairs was transformed into a new social space, for shared meals, groups activities and meeting with visitors	*“We had a gentleman who wouldn't really engage in therapy, but I gave him the job of watering the plants [in the new social area] every day and he started doing that and apparently he did better in therapy after the engagement sessions”. (Staff, Site 4, post).*
**Activity** – “Limited opportunities for patients to be active outside of therapy”	Activity boxes were provided for every four-bedded bay – items were chosen and boxes put together by co-design groups	*“We have huge gaps in the day where your patient’s doing nothing, they’re bored, they become institutionalised, so with these extras, like your volunteers coming in, you’ve got various groups, you’ve got your cooking group, your breakfast club, your lunch club, it just makes for a, well it’s a more positive experience.”* (Staff, site 2, post)
**Communication** – “Driven by structures and routines not enabling to activity”	A new webpage, information leaflet and posters were co-designed to emphasize activity and the importance of bringing in familiar and stimulating items from home, e.g. photos, games, electronic devices	*“I think the information leaflet’s quite good because it says, it tells you things like where the day room is and that you can go into the garden and things like that.…”.* (Carer, site 4, post)

Note: Space, Activity and Communication were priority areas agreed by patients, carers and staff at joint events. Quotes illustrate the impact of changes taken from interview and ethnographic observational data post implementation.

## Post implementation evaluation

### Quantitative findings

Behavioral mapping results post-implementation for patients meeting inclusion criteria and agreeing to consent the day before, were variable and overall levels of inactivity remained relatively high across all units. However, changes in the type of inactivity varied both within and across units (Table 9).

**Table 9.** Pre- and post-implementation behavioral mapping data

Site 1 Full EBCD	Pre-intervention, *n* = 9	Post-intervention, *n* = 7
No physical activity (%)	71	42
No cognitive activity (%)	68	72
No social activity (%)	58	52
Site 2 Full EBCD	Pre-intervention, *n* = 10	Post-intervention, *n* = 10
No physical activity (%)	65	66
No cognitive activity (%)	64	51
No social activity (%)	46	48
Site 3 Accelerated EBCD	Pre-intervention, *n* = 12	Post-intervention, *n* = 7
No physical activity (%)	50	56
No cognitive activity (%)	49	44
No social activity (%)	30	44
Site 4 Accelerated EBCD	Pre-intervention, *n* = 6	Post-intervention, *n* = 6
No physical activity (%)	61	65
No cognitive activity (%)	46	45
No social activity (%)	51	41

Footnote: Data from 60 observations taken at 10-min intervals for each participant per day recorded on three separate days 08.00–17.00 and 1300 and 20.00 including weekends. Mean percentage missing across sites =2.46% (range = 0.4–7.1%); 6031 total attempted observations; 142 missing (2.35%); 899 unobserved (14.91%); 845 sleeping (14.01%).

Response rates for PROM/PREMs were low varying from 12% to 38%. Cohorts who returned questionnaires were not atypical reporting levels of physical impairment, dependency, emotional and social limitations congruent with national and international stroke statistics. A full analysis from this data set is reported in our final report [https://njl-admin.nihr.ac.uk/document/download/2031395].

## Discussion

Overall, these findings support that using EBCD as an improvement approach was feasible and facilitated patients, carers, and staff collaborating to develop and implement multiple improvements to address patient activity in four stroke units. We also confirmed that the use of trigger films of patients’ experiences developed in two initial stroke units could be transferred to two further units and implemented within a shortened time frame using an accelerated form of EBCD (AEBCD).

This is the first report of using EBCD to address inactivity in stroke units. Stroke patients and carers played a significant role in eliciting changes through sharing experiences with each other and staff, highlighting priorities for improvement and engaging in the work of co-design. However, there was no signal of benefit to activity within quantitative data such as behavior mapping. This is despite staff perceptions of a more enabling environment and evidence of greater use of shared social spaces and number of activity opportunities in qualitative data, i.e. non-participant observations and interviews. Changes in priority areas environment (Space) and increased activity opportunities (Activity) for patients across each of the four participating stroke units were observed. Patient/staff interactions (Communication) achieved some ward level changes but were more challenging to initiate and sustain. These findings match previous studies which report challenges in changing the “nature” of communication towards enabling and personalized language.^22^

Despite an increase in the number of activity opportunities in all sites after EBCD cycles had completed, the impact on measured activity was equivocal. We believe this was due in part to the methodological constraints of case study research. We did not set out to compare individual patients before and after implementation of improvements because of the length of time taken to complete the (A)EBCD cycles, and we did not compare different cohorts within the same stroke units. Additionally, our findings could have been influenced by other factors. First, eligible patients for behavior mapping needed to provide consent the day before data collection, which meant we observed only a small number of patients and proportion of their activity behavior at any given time. Second, scheduled therapy sessions were recorded “unobserved” as were instances when a patient was not physically present but later found to have been at an outside café and thus active. Third, contextual issues such as staff shortages and the severity of disability of the inpatient caseload impacted on the activity opportunities we could record; we also recognize that not collecting detailed data on individual stroke severity was a limitation.

Overall, we believe that both EBCD and AEBCD are feasible and there were clear benefits of patients, carers, and staff identifying priorities and co-designing solutions. EBCD provided a facilitated, structured, participatory, and time limited process, fundamentally different to professionally-led – or externally driven – quality improvement initiatives in stroke. We believe the involvement of patients and carers increased the accountability of staff participants and the likelihood that planned changes would proceed. However, further research is warranted to fully understand how an increase in activity opportunities generated through CREATE can have the required impact on observed activity levels in patients on an inpatient stroke unit.

## Supplemental Material

sj-pdf-1-wso-10.1177_1747493020969367 - Supplemental material for Addressing inactivity after stroke: The Collaborative Rehabilitation in Acute Stroke (CREATE) study Click here for additional data file. Supplemental material, sj-pdf-1-wso-10.1177_1747493020969367 for Addressing inactivity after stroke: The Collaborative Rehabilitation in Acute Stroke (CREATE) study by Fiona Jones, Karolina Gombert-, Stephanie Honey, Geoffrey Cloud, Ruth Harris, Alastair Macdonald, Christopher McKevitt, Glenn Robert and David Clarke in International Journal of Stroke
