# Supplementary material for: Addressing inactivity after stroke: The Collaborative Rehabilitation in Acute Stroke (CREATE) study
Source: Int J Stroke. 2020 Nov 2;16(6):669–82. doi: 10.1177/1747493020969367 (PMC8366168; doi:10.1177/1747493020969367)
Supplement: sj-pdf-1-wso-10.1177_1747493020969367 - Supplemental material for Addressing inactivity after stroke: The Collaborative Rehabilitation in Acute Stroke (CREATE) study [file sj-pdf-1-wso-10.1177_1747493020969367.pdf]

Supplementary file 1

Pre-implementation- priorities driving changes

Space (selected quotes and observations)

The ward environment

"I often thought, on the stroke unit, it would have been nice to have a little group where they all could be interactive with each other instead of sitting staring at the walls…make the day spread out a bit better…I was always sitting watching things and thinking they could have a little group in the corner where they could be doing little things". (Carer, site 2, pre)

"The corridor is cluttered with table, trash bins etc. so we have to take turns [to pass]." "The corridor is cluttered with a hoist, 3 wheelchairs, various trolleys and trash bins."
(Field notes, site 1, pre)

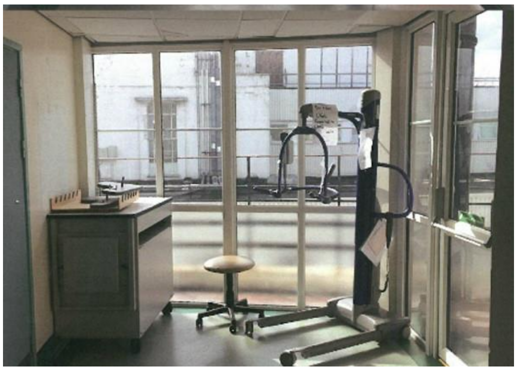

*Site **1 Equipment storage in corridors***

Supplementary file 1

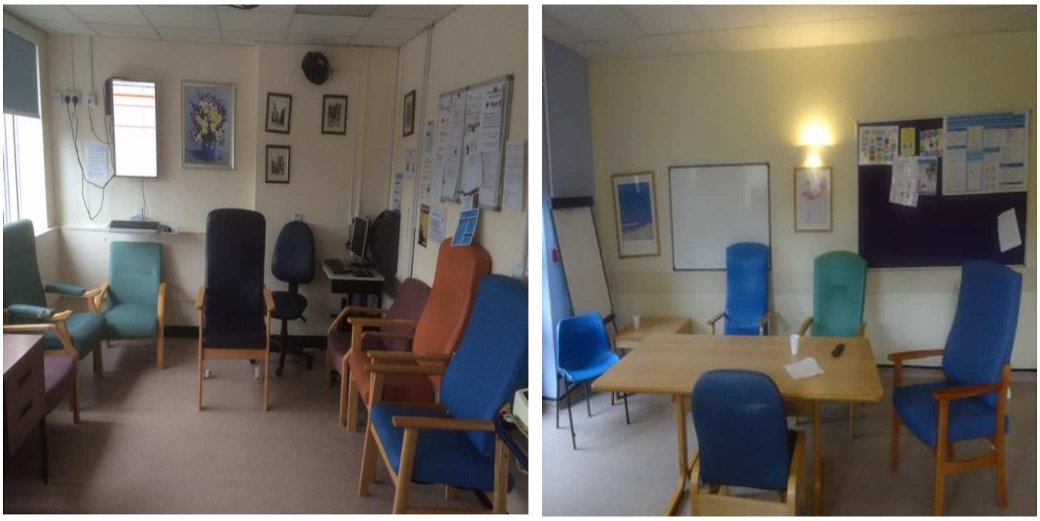

Site 2 and 3 **Day rooms used as meeting rooms for staff.**

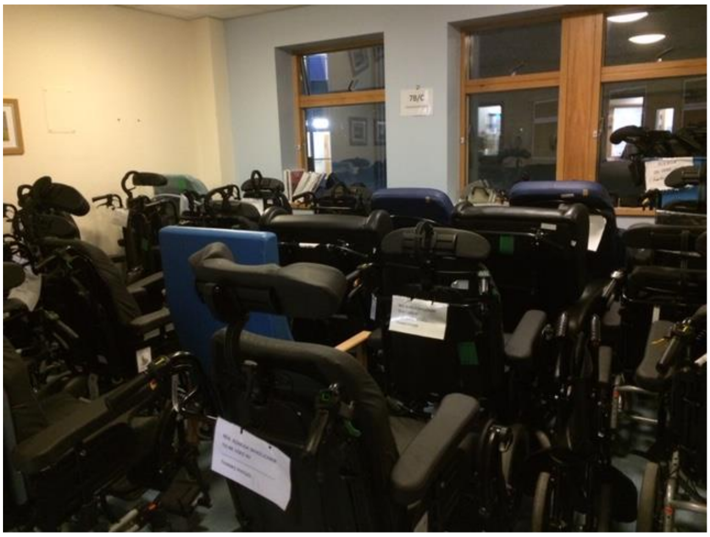

Day room at site 4 used for specialist chair and wheelchair storage.

The space around the bed

"On the movable table next to his bed, I see a little jar with water and a cup half-filled with orange juice, tissues and a blue folder like the ones used for patients' notes. On the bed side table there are two framed pictures –one is at the front and partially covers the other. The rest of the table is occupied by medical equipment." (Field notes, site 1, pre)

Supplementary file 1

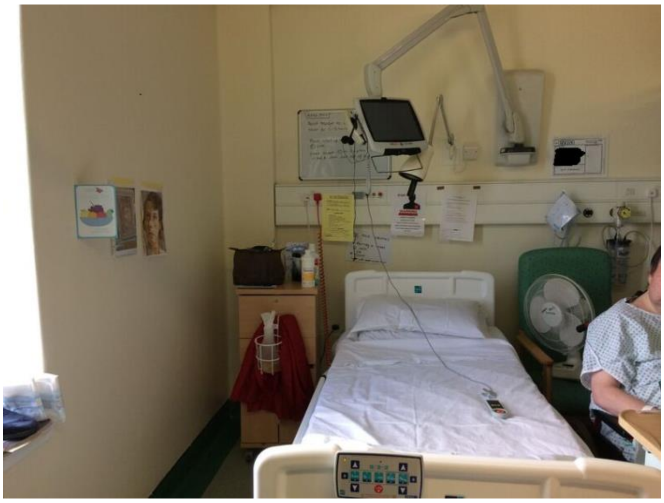

Figure 5 ***Typical bed space and décor in site 1 - described by patients and staff as cluttered and uninspiring***

Personalisation of bed space was rare- but led to tangible changes in the number of interactions

"I noted that the patient in bed 19 had a different bedside than other patients in the bay, as the family had brought in things that were important to her into the ward. She was keen to show me everything they had brought in, and felt it was immensely therapeutic to be able to look at pictures of family instead of the blank wall- *she said she had spent time gazing at them and thinking about each of them."* (Field notes site 1, pre)

Activity (selected quotes and observations)

"Nothing happens", says a Health Care Assistant (HCA). "Weekends are long", comments another HCA. "Weekdays are very busy – they [patients] have got everyone coming into their rooms. [At] weekends, they only see us. They get their care, obviously, but that's it. It's like – what happens now? Do I count down to Monday?" she says looking down at her wrist. (Field notes site 1 staff event)

"It would have been good to mix with other people, so you can get to know them" (Patient, site 3, pre)

"I would just love us to have a bit of money to use at our own discretion…on therapeutic items. Because you can get lots of things that you can do, , unilaterally, like one arm technique to do like

cross-stitch or something, like the stands and things that you can get and we don't have anything like that.[……] . We've got very, very dated equipment". (Staff, site 2, pre)

Communication (selected quotes and observations)

"*Hardly anyone else interacted with the patient. I heard no hand over of information about what the patient could do for themselves, or any aspect of their recovery, or activity."*

"I just found it horrible when no one even says a good morning, but they are discussing my condition. … Even down to saying things like "I am waiting for a letter from the council". It feels like your private life is not private at all. … Because I have had a stroke you lose everything, dignity, the ability to talk and walk, it feels horrible." (Patient, site 3, pre)

"She (HCA) just made the unbearable bearable, she really did. We all hated the weekends when she wasn't there, you're just bored anyway and there was mostly agency staff at weekends and, you know, it was just, we just so missed having her around. She just, you know, she used to say to us like "we'll laugh together and we'll cry together", you know, and she was just amazing." (Patient, site 3, post)

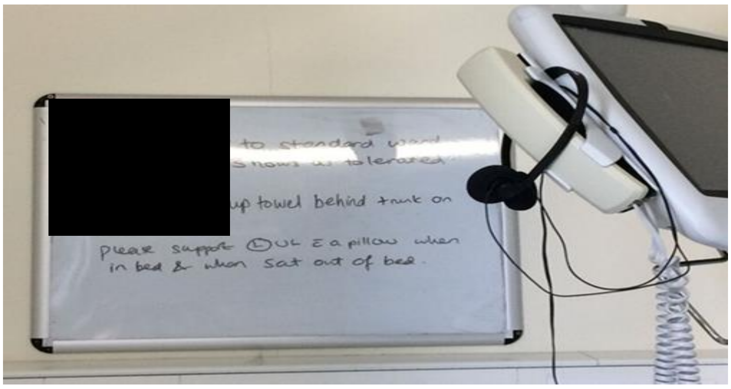

Figure 8 ***A typical method used in site 1 of conveying abbreviated clinical information written on white boards above beds***

Supplementary file 1

Impact of Changes

Post Implementation: Impact of changes

Space

"It is a picture of a beach and a cave, similar colours to the photograph from Cornwall that is on F's wall. He [family member] also shows the photo to F and tells her this is very similar. She smiles." (Field notes, site 1, post)

"He also uses his photo-hanger. There are get well cards and photos." (Field notes, site 1, post)

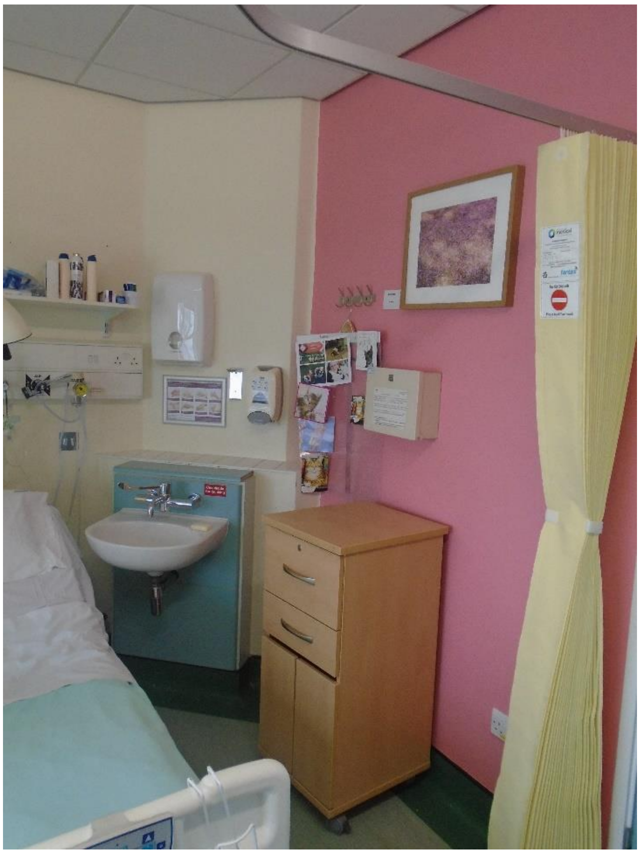

Showing one of the new colour schemes in a four bedded bay, clinical items were put on new shelves behind each bed, local art work on the wall, and a photo hanger at each bed (site1).

Supplementary file 1

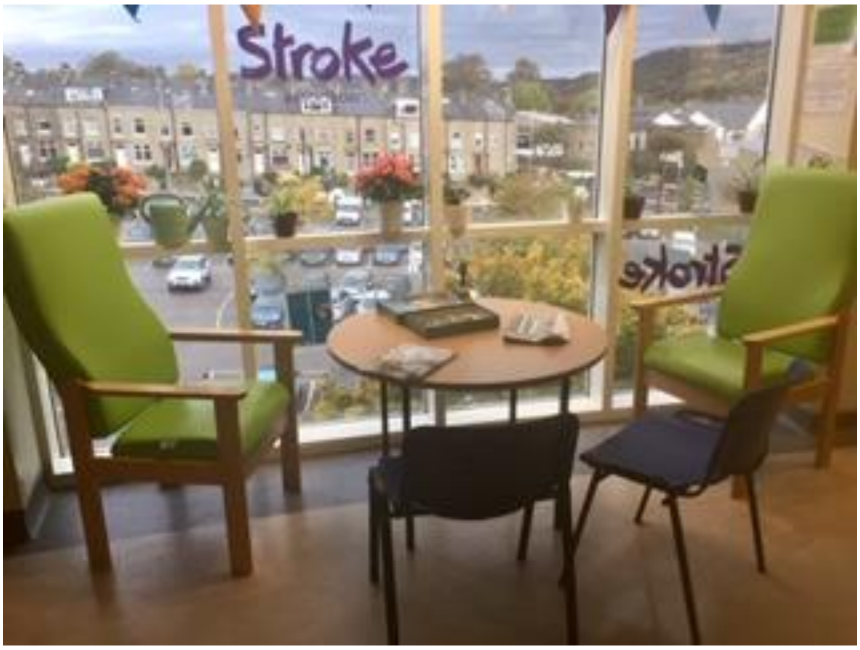

New repurposed seating area in site 4.

"We had a gentleman who was really disengaged, he wouldn't really engage in therapy, but I gave him the job of watering the plants [in the window area] every day and he started doing that and apparently he did better in therapy after the engagement sessions. One of the physios said 'I wasn't up for this woolly hippy stuff [CREATE] that you lot are up to but, I could see how it worked, it worked really well". (Staff, Site 4, post).

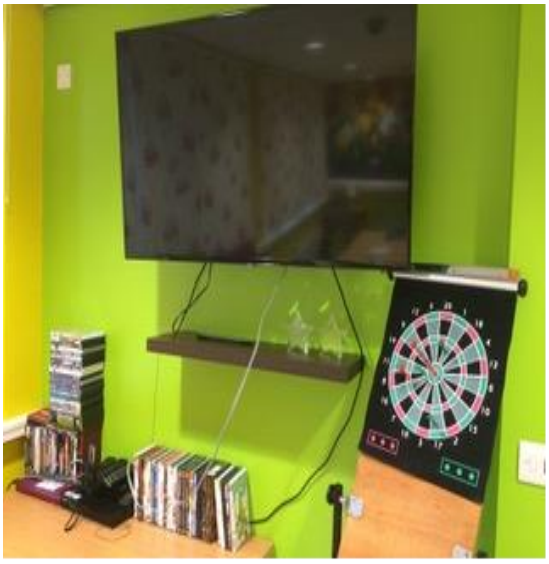

New social space (day room) used for scheduled and unscheduled activities in site 2.

Supplementary file 1

"I think it's great. Those rooms, that [ward D day] room is particularly good because, you know, when it was full of chairs we couldn't even go in there. Now it's, now it's a free resource we're lucky to have it, you know, and anything is better, yeah, I think it's great. I think those pagers are great, the iPads, you know, they're great on the stands, wonderful." (Patient, site 4, post)

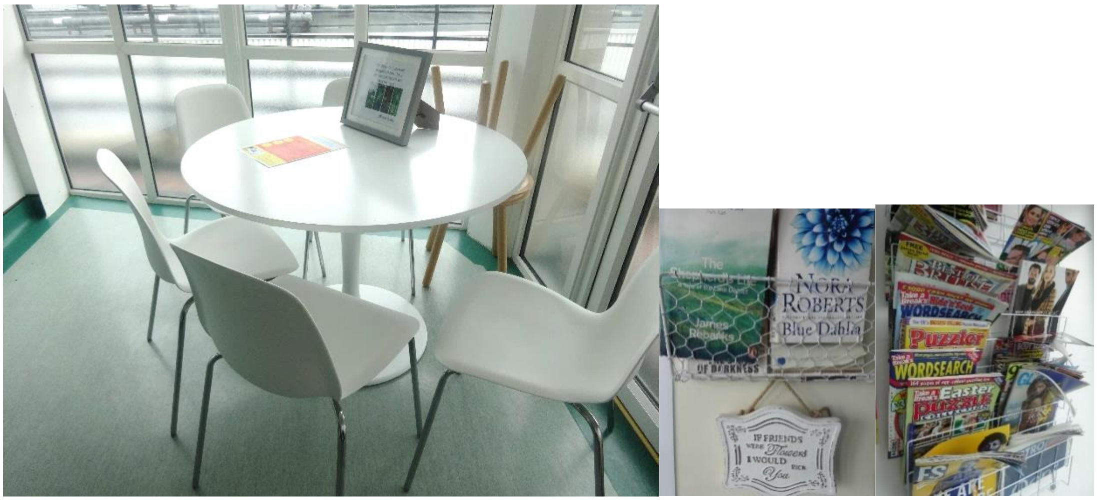

Figure 1 **New space in site 1 now an area for patients and families to meet and socialise.**

"I moved through the ward to the new social corner where I met patient F. who was doing a puzzle. Someone had brought it in for him, it was not his own he told me. He told me he thought the ward had become more inclusive over the time he was there. And he said he liked the social space". (Field notes, post, site 1)

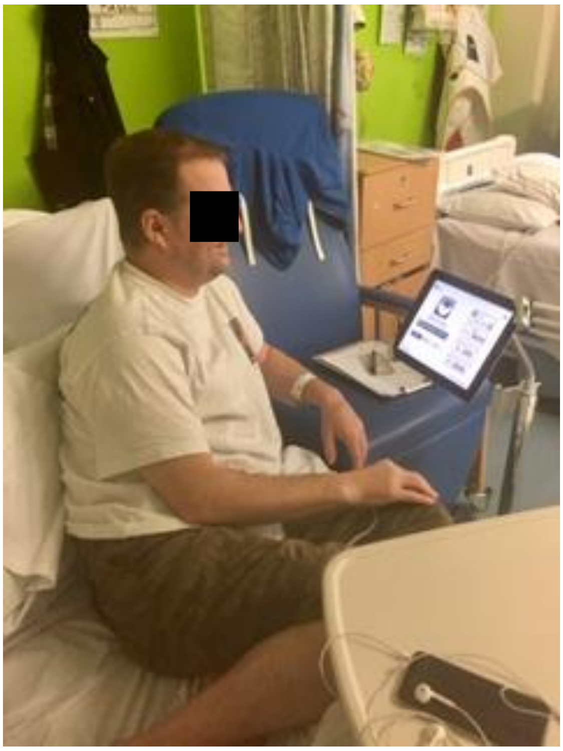

Figure 14 ***New mobile iPad stands used at the bedside in site 4.***

“I said that it is impressive as a fireman he must have saved many lives, and as an electrician bring light to people’s lives. Patient in bed 3 says “and electric shocks” and he laughs. We talk about their photos in the photo hanger. Patient in bed 4 has a holiday photo from Germany, a river, in his photo hanger. It is black and white. He said he went with his wife and it was a long time ago. Patient in bed 4 is very good with names, I realized this before. He knew the names of members of staff when man in bed 4 didn’t. He asked him again about someone’s name and he knew it. I tell him he is very good with names, the man in bed 3 agrees, says yes, isn’t he. I spent about 20 minutes with them. The patient in bed 3 asks me to pass him his blanket, it is red and cosy. He says it is very nice, he is a bit cold. His granddaughter brought it for him. His grandson used to play Rugby and they went to watch him on the weekends. He was very good he says. They say thank you and say it is very nice of me to put the programme on. They don’t want a newspaper to look at whilst waiting for the Rugby to start.” (Field notes, site 1, post).

Activities

Supplementary file 1

Changes s included: regular visits from therapy dogs (site 1 and 2); new activity boxes for every bay (site 1 and 3); increased structured group and individual therapeutic activities (all sites); increased volunteer and external group supported activities (music, gardening, massage, pet therapy, art and crafts, social Sunday lunches) (all sites).

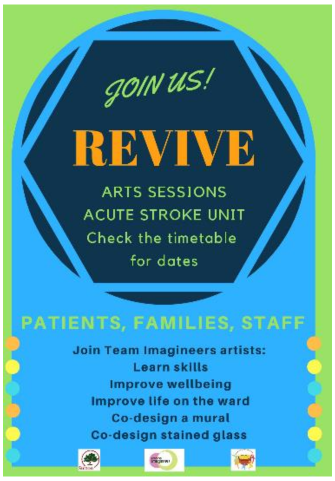

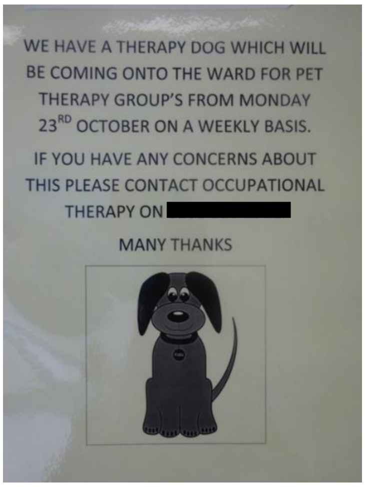

New activities (art groups and therapy dog) available in site 1.

"We have huge gaps in the day where your patient's doing nothing, they're bored, they become institutionalised, so with these extras, like your volunteers coming in, you've got various groups, you've got your cooking group, your breakfast club, your lunch club, it just makes for a, well it's a more positive experience isn't it, well I feel it is." (Staff, site 2, post)

"I do see evidence, psychological evidence [of being willing to engage in more activity]…and I also think patients are doing more now" (Patient and family members site 4 post)

"I think certain members of staff are really great at getting patients out of bed and really proactive with that and will look at what patients are signed up to do, whether it be therapy sessions or whether it be something like the Reading Group which has gone on timetables. And then there are other members of staff who don't look at the timetables at all" (Staff, site 3 post)

Communication

"I think the information leaflet's quite good because it says, it tells you things like where the day room is and that you can go into the garden and things like that. That new one, it's also on the wall outside the ward isn't it, the new one? Because I remember reading it there and I thought oh this is different from the one that used to be there when Mary was in". (Carer, site 4, post)

Supplementary file 1

"I think staff are thinking more about how to involve patients and get them talking and for myself I always go in now and make sure that I say hello to everyone, regardless, or introduce myself even if I'm not seeing that particular patient". (Staff, site 1, post)

"I am not sure if we have achieved much in terms of the basic communication day-to-day between the carers of the ward. Not the carers, the staff caring for the patients, because I think that's where we need an ongoing input. So, that's I think a big part, that's difficult to change and I'm not sure if it will change?" (Staff, site 3, post)
